# Paravalvular Leak After Transcatheter Aortic Valve Implantation: Haemodynamic Consequences, Clinical Impact, and Contemporary Assessment

**DOI:** 10.3390/jcdd13080382

**Published:** 2026-08-11

**Authors:** Mohamed Ali, Omran Abukhalaf, Mohammad Alkhalil

**Affiliations:** 1 Cardiothoracic Centre, Freeman Hospital, Newcastle-upon-Tyne NE7 7DN, UK; 2 Translational and Clinical Research Institute, Newcastle University, Newcastle-upon-Tyne NE1 7RU, UK

**Keywords:** transcatheter aortic valve implantation, paravalvular leak, haemodynamics, heart failure, VARC-3, aortic regurgitation, valve platforms

## Abstract

Paravalvular leak (PVL) remains an important limitation of transcatheter aortic valve implantation (TAVI), despite major advances in prosthesis design, imaging guidance, and implantation technique. Although the incidence of moderate and severe PVL has substantially declined with newer-generation valves, mild PVL continues to be frequently observed in contemporary practice and is increasingly recognised as clinically relevant. Multiple studies have demonstrated an association between even mild PVL and adverse outcomes, particularly heart failure (HF) hospitalisation, suggesting that conventional grading systems may underestimate its true haemodynamic burden. Beyond simple regurgitant volume, PVL is characterised by eccentric high-velocity flow, turbulence, impaired pressure recovery, and reduced haemodynamic efficiency. These effects may be particularly important in vulnerable phenotypes such as patients with heart failure with preserved ejection fraction (HFpEF), concentric left ventricular hypertrophy, or low-flow states, in whom small regurgitant volumes can result in disproportionate increases in filling pressures and symptomatic deterioration. This review summarises contemporary evidence regarding the prevalence, diagnosis, haemodynamic impact, and clinical consequences of PVL after TAVI. Differences across valve platforms, predictors of PVL, and current management strategies are also discussed. Particular emphasis is placed on the limitations of pressure-based assessment and the need for a patient-specific, flow-oriented approach aligned with contemporary endpoint definitions such as those proposed by VARC-3.

## 1. Introduction

Transcatheter aortic valve implantation (TAVI) has become an established treatment for severe aortic stenosis across the full spectrum of surgical risk, supported by multiple landmark randomised trials demonstrating outcomes comparable or superior to surgical valve replacement [1,2,3,4]. As procedural experience has evolved and device technology has improved, attention has increasingly shifted from procedural feasibility toward optimisation of long-term haemodynamic and clinical outcomes.

Paravalvular leak (PVL) emerged early as one of the principal limitations of TAVI. Initial-generation devices were associated with relatively high rates of residual aortic regurgitation due to incomplete sealing between the prosthetic frame and the native annulus [1,2]. Moderate or severe PVL was consistently linked to increased mortality and adverse cardiovascular outcomes [5,6]. Subsequent improvements in valve design, including external sealing skirts, enhanced radial force, and improved deliverability, have markedly reduced the incidence of clinically significant PVL [4,7,8].

Despite these advances, mild PVL remains common in contemporary TAVI practice and is increasingly recognised as more than a benign echocardiographic finding. Several observational studies and trial analyses have demonstrated an association between mild PVL and increased HF hospitalisation, impaired symptomatic recovery, and persistent haemodynamic abnormalities. These observations challenge the traditional assumption that mild regurgitation is haemodynamically trivial and suggest that conventional assessment methods may not adequately capture the true physiological burden of PVL [6,9].

Unlike central transvalvular regurgitation, PVL produces eccentric high-velocity jets through irregular paravalvular channels. This generates complex turbulent flow patterns, energy dissipation, impaired pressure recovery, and reductions in effective forward flow that are not fully reflected by conventional Doppler assessment or pressure-based haemodynamic indices. The haemodynamic impact of these abnormalities is likely to be influenced by the underlying ventricular phenotype, with patients exhibiting reduced ventricular compliance or low-flow states potentially being more susceptible to their physiological consequences.

This review summarises current evidence regarding the prevalence, diagnosis, haemodynamic consequences, and clinical implications of PVL following TAVI. Differences between valve platforms, predictors of PVL, and contemporary management strategies are also discussed, with particular emphasis on the growing importance of flow-oriented haemodynamic assessment.

## 2. Transvalvular Versus Paravalvular Regurgitation

Although both transvalvular and paravalvular regurgitation result in aortic insufficiency, their haemodynamic behaviour differs substantially.

Transvalvular regurgitation is typically central and related to leaflet dysfunction, malcoaptation, or structural degeneration. Flow tends to be more symmetric and predictable, allowing relatively consistent assessment using conventional echocardiographic parameters.

In contrast, PVL occurs at the interface between the prosthetic frame and native annulus. Regurgitant jets are frequently eccentric, irregular, and highly turbulent. As a result, the haemodynamic consequences of PVL may be less predictable and more difficult to quantify using conventional imaging techniques.

**Figure 1:**
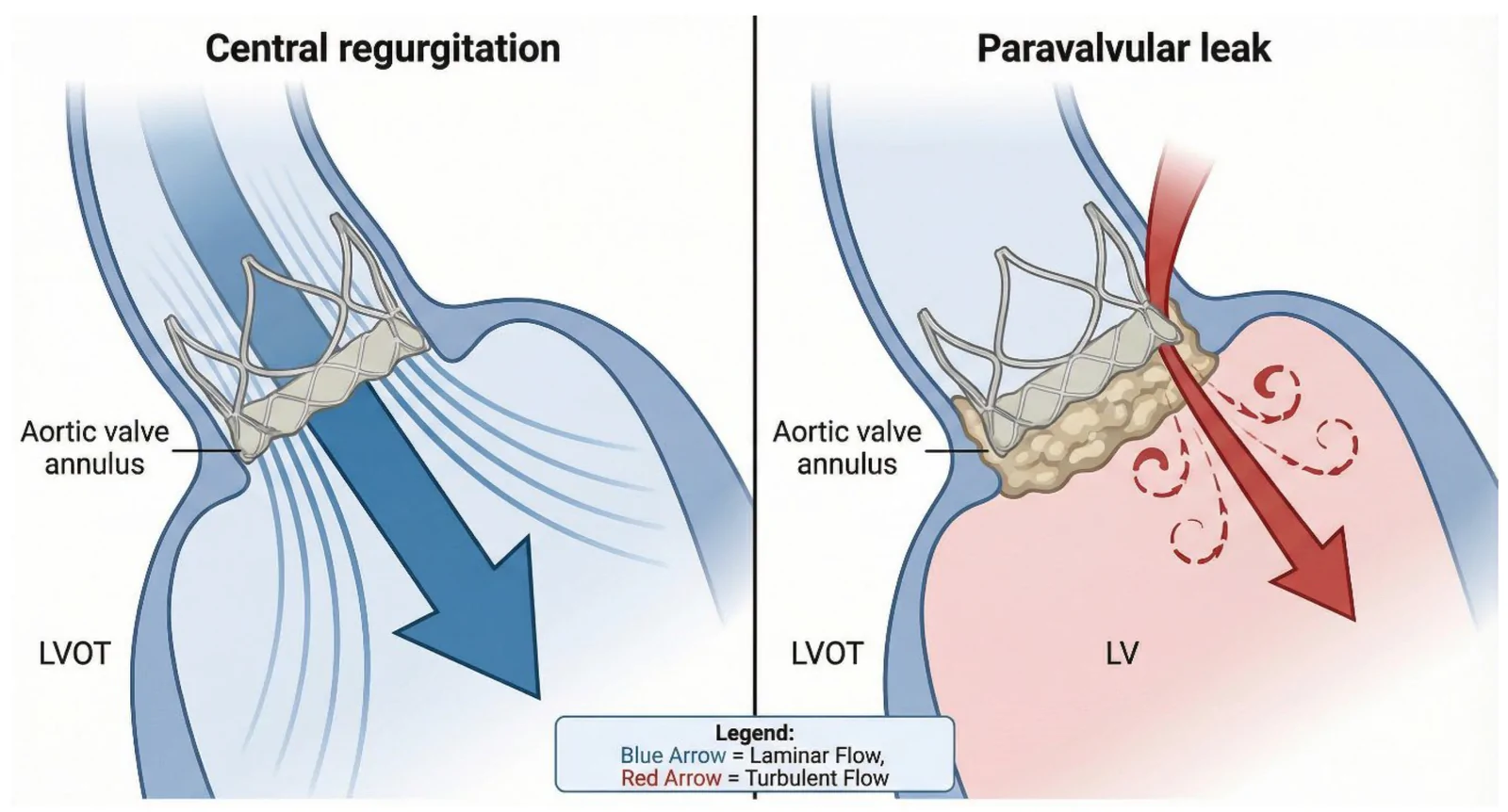
Mechanistic differences between central aortic regurgitation and paravalvular leak (PVL). In central aortic regurgitation (**left**), blood flows through the centre of the prosthetic valve, producing a relatively symmetrical, predominantly laminar regurgitant jet. In contrast, PVL (**right**) occurs through irregular channels between the prosthetic frame and the native annulus, generating eccentric high-velocity jets, turbulent flow, and energy dissipation. These differing flow characteristics may influence pressure recovery, effective forward flow, and the overall haemodynamic impact of residual regurgitation. Blue arrows indicate predominantly laminar flow, whereas red arrows indicate turbulent flow. (Abbreviations: LV, left ventricle; LVOT, left ventricular outflow tract).

This distinction is clinically important because conventional Doppler and pressure-based assessment tools were largely developed for central valvular regurgitation and may therefore underestimate the physiological burden imposed by PVL [9] (Figure 1).

## 3. Methods (Literature Search Strategy)

This narrative review was based on a comprehensive search of the published literature using PubMed/MEDLINE, Embase, and Google Scholar. Studies published in English from database inception to October 2025 were considered. Search terms included combinations of “transcatheter aortic valve implantation”, “TAVI”, “transcatheter aortic valve replacement”, “TAVR”, “paravalvular leak”, “paravalvular regurgitation”, “aortic regurgitation”, “haemodynamics”, “pressure recovery”, “heart failure”, “valve platform”, and “VARC-3”. Additional relevant publications were identified through manual screening of reference lists from selected articles and guideline documents. Priority was given to landmark randomised controlled trials, multicentre registries, contemporary observational studies, consensus documents, and guideline statements relevant to the diagnosis, haemodynamic assessment, clinical impact, and management of paravalvular leak after TAVI. The final selection of references was based on their clinical relevance and contribution to the objectives of this narrative review.

## 4. Prevalence and Temporal Trends of PVL After TAVI

The prevalence of PVL after TAVI varies according to valve platform, imaging modality, grading methodology, and patient anatomy. Representative rates of residual PVL reported in landmark trials and contemporary device studies are summarised in Table 1. Early-generation devices were associated with moderate or severe PVL in approximately 15–20% of procedures, reflecting limitations in frame conformability, sealing mechanisms, and procedural imaging [5,6].

Over the past decade, substantial improvements in prosthesis design have resulted in a marked decline in clinically significant PVL. Contemporary balloon-expandable and self-expanding valves incorporating external sealing skirts and enhanced annular conformability have reduced moderate or severe PVL rates to below 5% in most contemporary series [4,8].

However, while severe forms of PVL have become less frequent, mild PVL continues to be commonly observed, with reported rates ranging between 20% and 40% depending on the study population and imaging methodology [7,8,9,10]. Importantly, increasing evidence suggests that mild PVL may still carry prognostic significance, particularly with respect to HF hospitalisation and persistent symptomatic limitation [6,11].

The persistence of mild PVL reflects several unresolved anatomical and procedural challenges. Heavy annular calcification, asymmetric calcium distribution, elliptical annular geometry, and incomplete frame apposition may all contribute to residual paravalvular channels despite technically successful implantation. In addition, expanding TAVI into younger and lower-risk populations has increased the importance of minimising even small degrees of residual regurgitation [6,12].

These findings support the concept that PVL behaves more as a haemodynamic continuum than a purely categorical phenomenon. While traditional classifications distinguish mild, moderate, and severe regurgitation, the clinical impact of a given degree of PVL may vary substantially according to ventricular compliance, stroke volume reserve, and underlying haemodynamic phenotype [13].

## 5. Diagnosis and Severity Classification

### 5.1. Echocardiographic Assessment

Echocardiography remains the primary modality for assessing PVL after TAVI. Current grading relies predominantly on semi-quantitative parameters, including circumferential extent of regurgitation, jet width, vena contracta, colour Doppler characteristics, and flow reversal within the descending aorta [14,15,16].

However, accurate assessment of PVL can be challenging. Paravalvular jets are often eccentric, crescentic, irregular, or multiple, making conventional Doppler measurements less reliable than in central transvalvular regurgitation. Acoustic shadowing from valve frames and annular calcification may further impair visualisation. Consequently, inter-observer variability remains significant, particularly when differentiating between mild and moderate regurgitation.

### 5.2. Multimodality Imaging

Transoesophageal echocardiography (TOE) and cardiac computed tomography (CT) provide additional anatomical detail and may improve identification of PVL mechanism and localisation. CT imaging is particularly useful for assessing annular geometry, calcium burden, prosthesis expansion, and frame apposition.

More advanced imaging modalities, including four-dimensional flow magnetic resonance imaging (4D-flow MRI), have also demonstrated the ability to characterise complex regurgitant flow patterns and quantify energy dissipation. Although not currently routine in clinical practice, these techniques offer valuable mechanistic insights into the haemodynamic consequences of PVL [17,18].

### 5.3. Haemodynamic Indices in Procedural Assessment

Invasive haemodynamic measurements may complement imaging assessment during TAVI procedures. One of the most widely studied indices is the aortic regurgitation (AR) index, defined as:

$$\mathrm{AR}\mathrm{Index}=\frac{\mathrm{Diastolic}\;\mathrm{Blood}\;\mathrm{Pressure}-\mathrm{Left}\;\mathrm{Ventricular}\;\mathrm{End}\text{-}\mathrm{Diastolic}\;\mathrm{Pressure}}{\mathrm{Systolic}\;\mathrm{Blood}\;\mathrm{Pressure}}\times 100$$

Lower AR index values have been associated with increasing PVL severity and adverse clinical outcomes. An AR index below 25 has traditionally been considered suggestive of haemodynamically significant regurgitation and may support intraprocedural decision-making, including post-dilatation or valve repositioning, although it should be interpreted alongside imaging findings and the overall clinical context [19].

Nevertheless, reliance on pressure-based indices alone has important limitations. AR index measurements are influenced by loading conditions, vascular compliance, blood pressure, and ventricular stiffness. In patients with restrictive ventricular physiology or low-flow states, haemodynamic compromise may occur despite apparently acceptable pressure-derived indices.

### 5.4. Limitations of Current Classification Systems

Current grading systems predominantly focus on regurgitant severity rather than overall haemodynamic efficiency. Conventional approaches do not adequately account for turbulence, energy dissipation, impaired pressure recovery, or reductions in effective forward flow.

**Figure 2:**
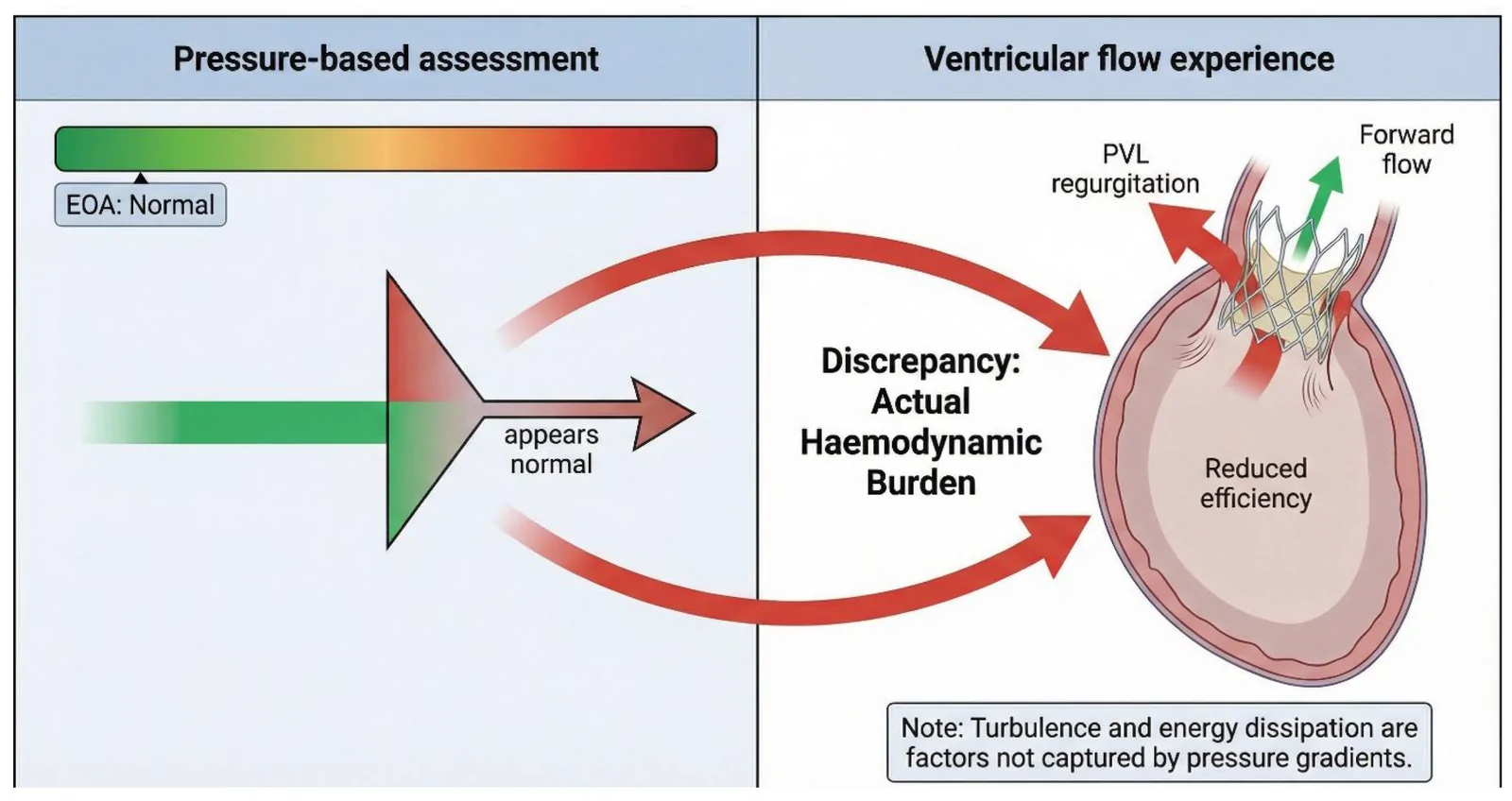
Limitations of pressure-based haemodynamic assessment in PVL. Conventional pressure-based indices and Doppler-derived measurements may suggest satisfactory valve haemodynamics despite the presence of residual PVL. However, turbulence, energy dissipation, and reductions in effective forward flow may contribute to a greater physiological burden than is reflected by pressure-based assessment alone. This figure illustrates a conceptual model highlighting the potential discrepancy between conventional haemodynamic assessment and the overall physiological impact of residual PVL. (Abbreviations: EOA, effective orifice area).

As a result, standard classifications may underestimate the physiological burden of PVL, particularly in vulnerable ventricular phenotypes. This may help explain why apparently mild PVL continues to demonstrate clinically relevant associations with HF hospitalisation and persistent symptoms [9,15,16] (Figure 2).

The Valve Academic Research Consortium-3 (VARC-3) consensus provides standardised endpoint definitions for TAVI studies, including updated definitions of bioprosthetic valve dysfunction and valve-related complications. Although these criteria improve consistency in clinical trial reporting and facilitate comparison between studies, they do not fully account for the physiological impact of residual PVL or differences in ventricular phenotype. Consequently, integrating conventional endpoint definitions with patient-specific haemodynamic assessment may provide a more comprehensive evaluation of residual PVL [20].

## 6. Haemodynamic Consequences of PVL

### 6.1. Flow Characteristics and Energy Dissipation

The haemodynamic behaviour of PVL differs substantially from that of central transvalvular regurgitation. PVL typically occurs through narrow, irregular paravalvular channels that generate high-velocity eccentric jets and complex turbulent flow patterns.

This turbulence is likely to contribute to energy dissipation and haemodynamic inefficiency, reducing the proportion of total stroke volume that contributes to effective systemic forward flow. Consequently, patients may exhibit apparently satisfactory transvalvular gradients while still experiencing impaired ventricular unloading and persistent symptoms [9,21].

### 6.2. Pressure Recovery and Effective Forward Flow

Pressure recovery refers to the partial reconversion of kinetic energy into pressure distal to the valve. In the presence of PVL, disturbed and turbulent flow may impair the pressure recovery process, reducing overall haemodynamic efficiency.

Importantly, Doppler-derived gradients may therefore underestimate the true haemodynamic burden experienced by the ventricle. Patients may demonstrate acceptable echocardiographic valve gradients despite persistent reductions in effective forward stroke volume and ongoing ventricular stress.

**Figure 3:**
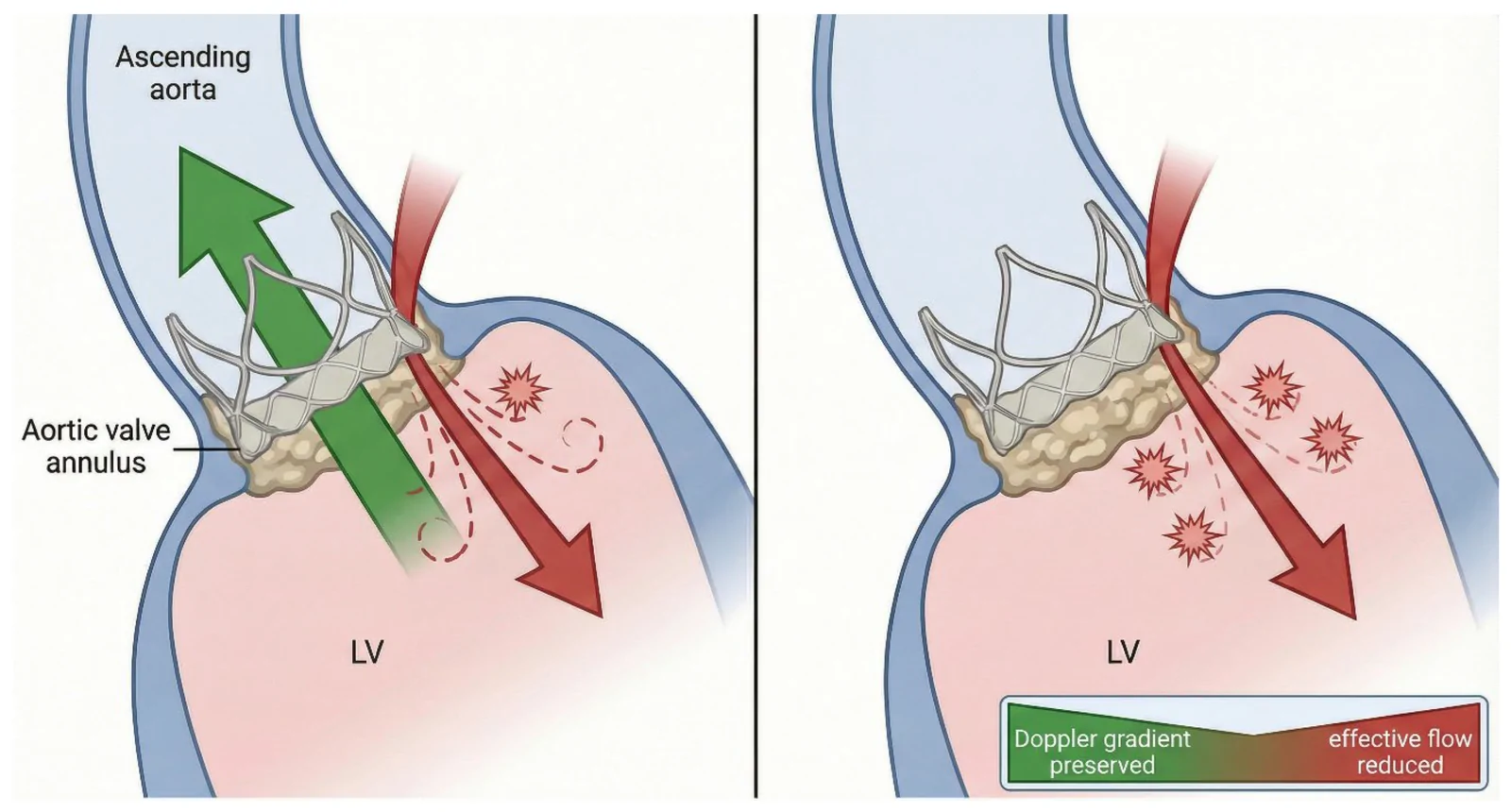
Impact of PVL on pressure recovery and haemodynamic efficiency. Residual PVL generates eccentric regurgitant flow through irregular paravalvular channels, producing turbulence and energy dissipation. Although conventional Doppler assessment may suggest satisfactory valve haemodynamics, disturbed flow may impair pressure recovery and reduce effective forward flow. Consequently, patients with similar echocardiographic grades of PVL may experience different physiological and clinical consequences. This figure illustrates a conceptual model rather than a validated physiological pathway. (Abbreviations: LV, left ventricle).

This concept may partly explain the apparent discordance observed in some patients between favourable procedural haemodynamics and limited clinical improvement [9,21] (Figure 3).

### 6.3. Left Ventricular and Pulmonary Haemodynamics

Residual PVL leads to regurgitant volume loading of the left ventricle, increasing LV end-diastolic pressure and impairing effective forward output. In susceptible patients, even relatively small regurgitant volumes may result in disproportionate increases in filling pressures.

Secondary elevation in left atrial pressure may subsequently contribute to pulmonary venous congestion and pulmonary hypertension, thereby increasing symptomatic burden and HF risk.

In contrast to surgical aortic valve replacement, where complete annular debridement often facilitates more complete elimination of regurgitation, residual PVL following TAVI may contribute to persistent haemodynamic abnormalities despite technically successful valve implantation.

### 6.4. Differential Haemodynamic Vulnerability

The clinical impact of PVL may be influenced by ventricular phenotype.

Patients with HFpEF or concentric hypertrophic remodelling exhibit reduced ventricular compliance and impaired diastolic reserve. In these patients, small increases in regurgitant volume may produce marked rises in LV filling pressure and pulmonary congestion, potentially limiting haemodynamic recovery despite technically successful TAVI.

Similarly, patients with low-flow, low-gradient aortic stenosis represent a particularly vulnerable group. Because forward stroke volume reserve is already limited, residual PVL may further reduce effective systemic output, potentially worsening exercise tolerance and impairing symptomatic improvement.

**Figure 4:**
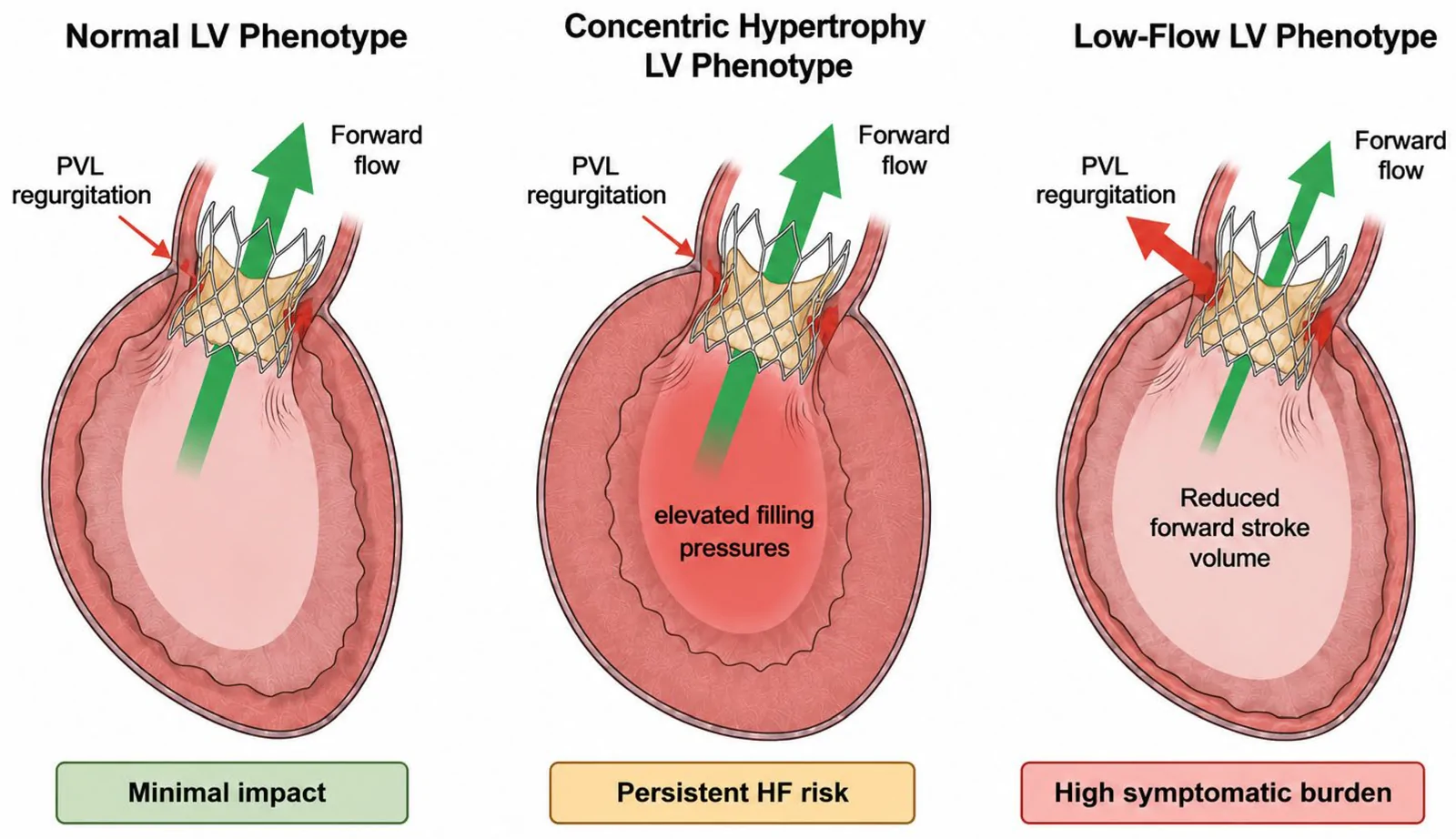
Proposed influence of ventricular phenotype on the haemodynamic impact of PVL. The physiological consequences of residual PVL may vary according to the underlying ventricular phenotype. Patients with preserved ventricular compliance may tolerate small degrees of PVL with minimal haemodynamic impact, whereas those with concentric hypertrophy or low-flow physiology may experience disproportionate increases in filling pressures, reduced effective forward flow, and persistent symptoms despite similar echocardiographic grades of regurgitation. This figure illustrates a conceptual model rather than a validated physiological pathway. (Abbreviations: LV, left ventricle; HF, heart failure).

These observations align closely with the increasing emphasis placed by VARC-3 on HF hospitalisation and functional recovery as clinically meaningful post-TAVI endpoints [20] (Figure 4).

## 7. Clinical Outcomes Associated with PVL

A consistent relationship has been demonstrated between PVL severity and adverse clinical outcomes following TAVI.

Moderate and severe PVL have repeatedly been associated with increased mortality, recurrent HF, and impaired long-term outcomes [5,6]. More recently, attention has shifted toward the impact of mild PVL, which remains common even with contemporary devices.

Several observational studies and trial analyses have reported an association between mild PVL and increased HF hospitalisation, persistent symptoms, and reduced quality-of-life improvement [6,9,11,22,23]. Although the absolute haemodynamic burden of mild regurgitation may appear limited, its cumulative physiological effects may become clinically relevant in vulnerable patient populations.

Importantly, these findings have been reported across different valve platforms and patient risk profiles, suggesting that the adverse effects of PVL are not solely device-specific but instead reflect broader haemodynamic consequences related to ineffective forward flow and ventricular loading.

These observations further support the need to move beyond purely categorical grading systems and toward a more integrated assessment incorporating ventricular phenotype, flow efficiency, and clinical context.

## 8. Device Platform Comparisons

The incidence of PVL varies across transcatheter valve platforms and is influenced by differences in frame design, radial force, annular conformability, and sealing mechanisms.

Balloon-expandable valves have traditionally demonstrated lower rates of PVL, partly due to greater radial force and the incorporation of external sealing skirts. Self-expanding valves offer advantages in conformability and repositionability but historically exhibited higher rates of mild residual regurgitation [4,7,10].

Recent-generation devices have substantially narrowed these differences. In the prospective PORTICO NG study, the Navitor system demonstrated low rates of moderate or severe PVL at one year [8]. Similarly, early multicentre experience with the Evolut FX system demonstrated excellent haemodynamic performance and very low rates of moderate or severe PVL at 30 days [24].

Nevertheless, mild PVL continues to occur across all contemporary valve systems. Interpretation of comparative data is further complicated by differences in patient selection, annular anatomy, imaging methodology, and endpoint definitions across studies.

The impact of skirtless or alternative frame designs also remains less clearly defined. While some platforms may offer procedural advantages in specific anatomies, uncertainty persists regarding long-term haemodynamic performance and residual regurgitation.

Overall, although modern device design has significantly improved PVL outcomes, no single platform completely eliminates residual regurgitation, reinforcing the importance of patient-specific procedural planning and implantation technique.

## 9. Predictors of PVL

PVL is multifactorial and results from the interaction between patient anatomy, prosthesis characteristics, and procedural factors.

Among anatomical predictors, heavy annular calcification appears particularly important. Asymmetric or bulky calcium distribution may impair complete prosthesis expansion and prevent optimal sealing against the native annulus. Elliptical annular geometry and severe leaflet calcification may further increase the risk of residual paravalvular channels [25].

Procedural factors also play a major role. Undersizing, valve malposition, incomplete expansion, and suboptimal implantation depth have all been associated with increased PVL risk [9,26].

Although smaller annular dimensions may increase procedural complexity, annular calcification pattern often appears more predictive of PVL than annular size alone [27].

Bicuspid aortic valve anatomy may further increase the risk of PVL because eccentric annular geometry and asymmetric leaflet calcification can impair prosthesis expansion and complete frame apposition. Although outcomes with newer-generation devices continue to improve, careful pre-procedural CT assessment and appropriate device selection remain particularly important in this subgroup [12].

Patient-specific haemodynamic characteristics may additionally influence the clinical impact of PVL. Patients with impaired ventricular compliance, HFpEF physiology, or low-flow states may be less tolerant of residual regurgitation and may experience greater symptomatic deterioration despite relatively mild PVL.

## 10. Management Strategies

### 10.1. Prevention and Procedural Planning

Prevention remains the most effective strategy for minimising PVL.

Key preventive measures include:

- meticulous pre-procedural CT assessment;
- accurate annular sizing;
- evaluation of calcium distribution and annular geometry;
- appropriate device selection tailored to patient anatomy [12].

Careful procedural planning has become increasingly important as TAVI expands into younger and lower-risk populations with longer life expectancy.

### 10.2. Intra-Procedural Optimisation

Intra-procedural optimisation strategies include:

- balloon post-dilatation;
- valve repositioning when feasible;
- optimisation of implantation depth;
- integration of imaging and invasive haemodynamic assessment.

Post-dilatation may improve prosthesis expansion and sealing but must be balanced against the risk of annular injury, stroke, or conduction disturbance [12].

### 10.3. High-Risk Haemodynamic Phenotypes

Patients with HFpEF, concentric hypertrophy, pulmonary hypertension, or low-flow states may warrant a lower threshold for optimisation because even small degrees of residual PVL may have clinically significant consequences.

In such patients, procedural precision and device selection become particularly important.

### 10.4. Post-Procedural Management

Post-procedural management may include:

- optimisation of guideline-directed HF therapy;
- repeat haemodynamic and imaging assessment;
- consideration of valve-in-valve implantation [28];
- percutaneous PVL closure in selected cases [12,29].

Although uncommon, haemolytic anaemia is a recognised complication of residual PVL after TAVI. Management depends on the severity of haemolysis and may include conservative treatment, percutaneous PVL closure, repeat valve intervention, or surgery in selected severe cases.

Future approaches may increasingly incorporate more sophisticated flow-based haemodynamic assessment to identify patients at highest risk despite apparently mild residual regurgitation.

## 11. Future Directions: Towards a Flow-Oriented Assessment of Paravalvular Leak

Despite substantial advances in prosthesis design and implantation technique, assessment of residual paravalvular leak remains largely based on imaging-derived severity grading and pressure-based haemodynamic indices. While these approaches remain fundamental to contemporary clinical practice, they do not fully account for the considerable variability in clinical outcomes observed among patients with apparently similar degrees of regurgitation. This suggests that the physiological burden of PVL is determined by more than regurgitant severity alone and reflects a complex interaction between anatomical characteristics, haemodynamic performance, and the patient’s underlying cardiovascular phenotype.

The tolerance of residual PVL is likely influenced by factors including ventricular compliance, stroke volume reserve, ventricular–arterial coupling, and pulmonary haemodynamics. Consequently, a small eccentric paravalvular jet may be well tolerated in one patient yet result in persistent symptoms, recurrent heart failure, or impaired functional recovery in another despite an identical echocardiographic grade. Such variability highlights the limitations of interpreting PVL according to conventional severity classifications alone and supports a broader assessment that considers the overall haemodynamic impact of residual regurgitation.

**Figure 5:**
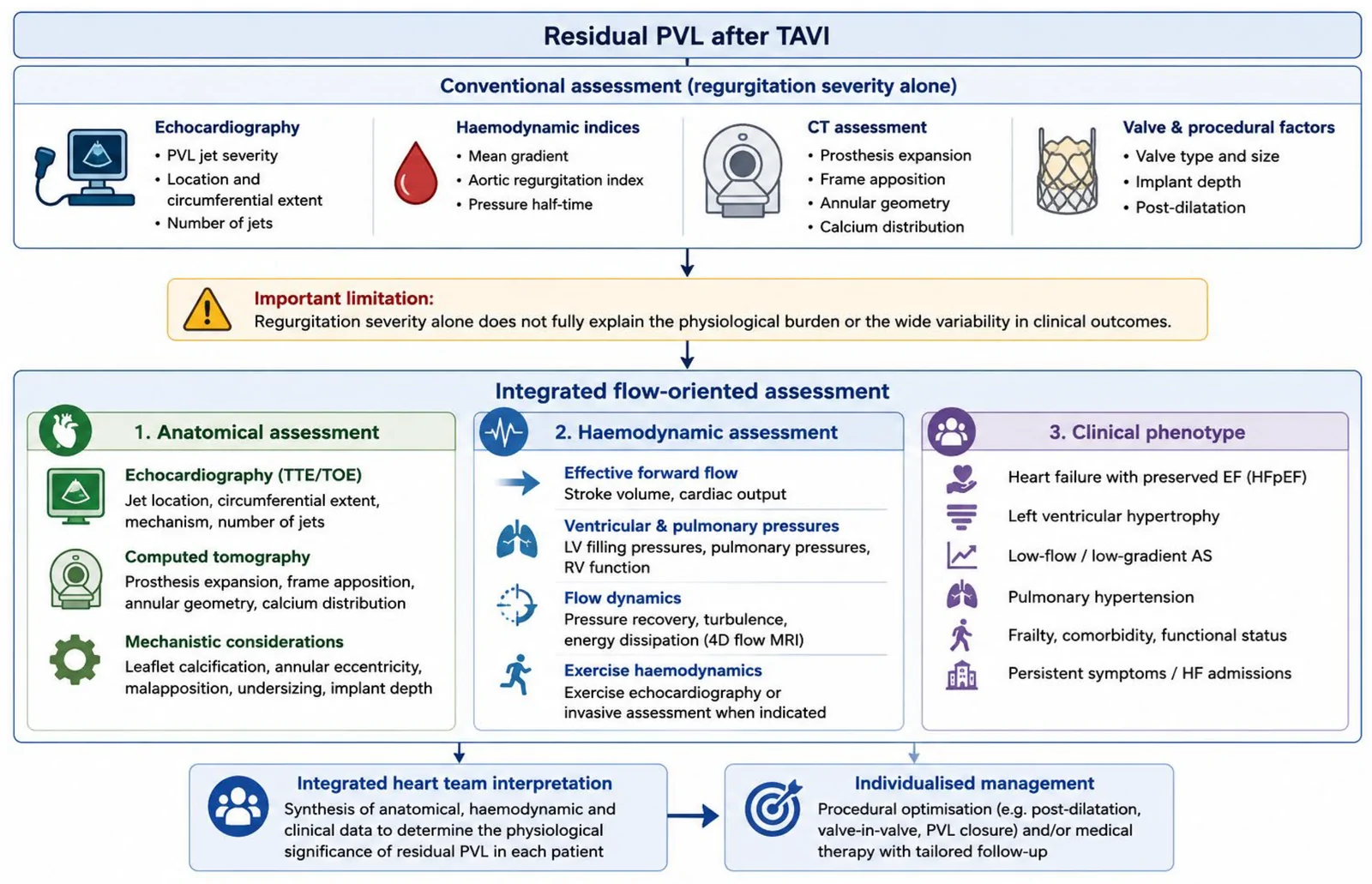
Towards a flow-oriented assessment of PVL after TAVI. Conventional assessment of residual PVL relies predominantly on regurgitation severity, imaging findings, and pressure-based haemodynamic indices. This conceptual framework proposes an integrated assessment combining anatomical evaluation, haemodynamic performance, and patient clinical phenotype to better characterise the physiological burden of residual PVL and support individualised clinical decision-making. The framework is intended to stimulate future research rather than represent a validated clinical algorithm. (Abbreviations: AS, aortic stenosis; CT, computed tomography; HF, heart failure; HFpEF, heart failure with preserved ejection fraction; LV, left ventricle; MRI, magnetic resonance imaging; RV, right ventricle; TTE, transthoracic echocardiography; TOE, transoesophageal echocardiography).

Figure 5 illustrates a conceptual approach in which assessment of residual PVL integrates three complementary domains: anatomical assessment, haemodynamic assessment, and clinical phenotype. Anatomical assessment remains the cornerstone of PVL evaluation. Comprehensive transthoracic echocardiography should define the location, circumferential extent, and mechanism of regurgitation, while transoesophageal echocardiography and cardiac computed tomography provide additional information regarding prosthesis expansion, frame apposition, annular geometry, and calcium distribution when uncertainty persists. Together, these modalities define the structural substrate responsible for residual leak and may help identify potentially correctable procedural factors.

Haemodynamic assessment should extend beyond transvalvular gradients and the aortic regurgitation index alone. Additional consideration should be given to parameters that better reflect effective forward flow and ventricular performance, including stroke volume, left ventricular filling pressures, pulmonary haemodynamics, and exercise physiology where clinically appropriate. Emerging techniques such as four-dimensional flow magnetic resonance imaging and invasive haemodynamic assessment may further characterise flow disturbance, pressure recovery, and energy dissipation, providing mechanistic insights that are not captured by conventional grading systems. Although these modalities are not currently incorporated into routine clinical practice, they represent promising tools for future evaluation of the physiological burden of PVL.

Interpretation of residual PVL should also be individualised according to the patient’s clinical phenotype. Patients with heart failure with preserved ejection fraction, concentric left ventricular hypertrophy, pulmonary hypertension, or low-flow states may be particularly susceptible to even modest reductions in effective forward flow and therefore experience disproportionate haemodynamic and clinical consequences despite apparently mild residual regurgitation. Integrating anatomical findings, haemodynamic performance, and patient phenotype may therefore provide a more comprehensive assessment than regurgitation severity alone and help explain the discordance frequently observed between imaging findings and clinical outcomes.

The conceptual approach presented in Figure 5 is intended to stimulate further research rather than serve as a validated clinical algorithm. As TAVI continues to expand into younger and lower-risk populations, greater emphasis will be placed on optimising long-term haemodynamic performance rather than simply reducing procedural complications. Future prospective studies should determine whether integrating anatomical imaging, haemodynamic performance, and patient phenotype improves risk stratification, guides procedural optimisation, and predicts long-term clinical outcomes more accurately than current grading systems.

## 12. Conclusions

Paravalvular leak remains a common finding after contemporary TAVI despite substantial advances in valve technology and implantation technique. Although the incidence of moderate and severe PVL has declined significantly, mild residual regurgitation continues to occur frequently and is increasingly recognised as clinically relevant.

The haemodynamic impact of PVL extends beyond regurgitant volume alone. Turbulence, energy dissipation, impaired pressure recovery, and reductions in effective forward flow may contribute to persistent ventricular stress despite apparently acceptable conventional haemodynamic indices.

Importantly, the physiological consequences of PVL are strongly influenced by ventricular phenotype. Patients with HFpEF, concentric hypertrophy, or low-flow states appear particularly vulnerable to even small degrees of residual regurgitation.

These findings highlight the limitations of purely pressure-based assessment and support a more comprehensive, patient-specific approach integrating imaging, haemodynamics, and clinical phenotype. Such an approach is increasingly aligned with contemporary endpoint definitions emphasising HF hospitalisation and functional recovery following TAVI.

## Figures and Tables

**Table 1:** Landmark Trials Reporting PVL at 1 Year After TAVI.

Study	Population	Valve	Risk	Moderate/Severe PVL	Mild PVL
PARTNER 2 [3]	Intermediate	Sapien XT/3	Intermediate	~3–4%	~20–30%
PARTNER 3 [4]	Low risk	Sapien 3	Low	<1%	~25–30%
SURTAVI [10]	Intermediate	CoreValve/Evolut	Intermediate	~5–6%	~35–45%
Evolut Low Risk [7]	Low risk	Evolut R/PRO	Low	~3–5%	~30–40%
Navitor IDE [8]	Mixed	Navitor	Mixed	~1–2%	~20–25%

## Data Availability

No new data were created or analyzed in this study. Data sharing is not applicable to this article.
